# Relationship between energy balance-related behaviors and personal and family factors in overweight/obese primary school students aged 10–12 years in China: a cross-sectional study

**DOI:** 10.1186/s12889-022-14238-x

**Published:** 2022-10-27

**Authors:** Shicheng Zhang, Haining Gao, Ying Cui, Xin Wang, Wenshuo Cao, Qian Ding, Bo Chang

**Affiliations:** 1 Liaoning Normal University, Dalian, 116029 Liaoning China; 2 Shenyang Sport University, Shenyang, 110115 Liaoning China

**Keywords:** Overweight/obesity, School-aged children, Energy balance-related behavior, Influencing factors

## Abstract

**Background:**

Increasing rates of childhood obesity worldwide are a serious threat to the health of school-aged children. Unhealthy behavioral habits are modifiable factors in the control of childhood obesity, and personal and family factors are key influencing factors of behavioral habits in school-aged children. This study assessed the relationship between overweight/obesity, energy balance-related behaviors (EBRB), and their influencing factors in school-aged children.

**Methods:**

This cross-sectional survey included 4412 primary school-aged (10–12 years) students who underwent body tests and were selected through stratified sampling in the Northeast, North, Northwest, and Southwest regions of China from March to July 2021. Independent sample t test was used to compare differences between behaviors and influencing factors of energy balance among overweight/obesity and normal weight students. Logistic regression analysis was used to assess the influence of EBRB on body shape. Multiple linear regression was used to assess the influence of personal and family factors on EBRB effects.

**Results:**

Compared with normal-weight students, number of breakfasts consumed per week by overweight/obese students was significantly lower (*p* < 0.01), and weekly screen-viewing time was significantly longer (*p* < 0.01). Overweight/obese students’ health beliefs, parental subjective norms, parental modelling, parental practices, and home availability scores increased significantly in terms of beverage consumption behavior (*p* < 0.01 or *p* < 0.05). Attitude, health beliefs, self-efficacy, parental subjective norms, and parental support scores decreased significantly in terms of breakfast consumption (*p* < 0.01 or *p* < 0.05). Health belief scores on physical activity increased significantly (*p* < 0.01), while preference and autonomy scores decreased significantly (*p* < 0.01). Health beliefs, parental subjective norms, and parental practices scores of screen-viewing activities increased significantly (*p* < 0.01 or *p* < 0.05). Breakfast consumption (odds ratio [OR]: 0.911; 95% confidence interval [CI]: 0.870–0.954) and screen-viewing activities (OR:1.055; 95% CI: 1.030–1.080) correlated negatively and positively with overweight/obesity, respectively. The main influencing factors of breakfast behavior in overweight/obese students were self-efficacy (0.14), preference (0.11), attitude (0.07), home availability (0.18), and parent modelling (0.09); those for screen-viewing behavior were preference (0.19), self-efficacy (− 0.15), parental practices (0.13), and parental subjective norm (0.12).

**Conclusions:**

Irregular breakfast consumption and excessive screen-viewing time are key EBRB associated with overweight/obesity among these Chinese participants. Their unhealthy breakfast consumption and screen-viewing activities result from a combination of personal and family factors.

**Supplementary Information:**

The online version contains supplementary material available at 10.1186/s12889-022-14238-x.

## Background

Overweight/obesity not only affects the growth and development of school-aged children, but also increases the risk of developing metabolic syndrome, cardiovascular disease, and even cancer in adulthood [1–3]. According to statistics published by the World Health Organization, the number of overweight and obese children aged 5–19 years had exceeded 340 million worldwide, representing more than 18% of the total global population of school-aged children [4]. China is the most populous country in the world, and the total number of obese people has continued to grow rapidly in the past 40 years, ranking first in the world [5]. The 2020 Report on Nutrition and Chronic Diseases of Chinese Residents showed that the overweight and obesity rate of children and adolescents in China had rapidly increased from 6.6% in 2002 to 19.0% in 2020 [6]. Thus, overweight and obesity have become a prominent health problem in school-aged children in China.

The root cause of overweight/obesity is a disrupted energy balance [7]. Unhealthy eating behaviors increase energy intake, while sedentary behaviors reduce energy consumption. The combined effect of the two biases the energy balance toward a positive balance. Long-term positive energy balance causes excessive accumulation of lipids in the body, resulting in overweight/obesity [8]. Studies have shown that overweight/obesity in school-aged children is associated with some unhealthy behaviors, including drinking sugar-sweetened beverages [9], skipping breakfast [10], low levels of physical activity [11], and prolonged screen-viewing behaviors [12]. These behaviors that can affect energy balance are referred to as energy balance-related behaviors (EBRB) [13]. EBRB are affected by a variety of factors, among which the child’s individual and family factors are crucial [14]. These include school-aged children’s own attitudes, health beliefs, preference, and self-efficacy [15–17], as well as parental subjective norms [18], parent modeling [19], parental practices [20], and encouragement or parental support [21].

Current research on EBRB has focused on the association of certain behaviors (drinking of sugar-sweetened beverages, skipping breakfast, low levels of physical activity, and prolonged screen-viewing behaviors) with overweight/obesity in school-aged children. There are few studies that consider multiple behaviors at the same time. “European Energy balance Research” is the first to conduct a comprehensive study of overweight/obesity and EBRBs in school-aged children aged 10–12 and their influencing factors. Study found that school-aged children in different countries have unhealthy ERBR to varying degrees [22] and these behaviors are affected by both individual and family factors [14, 23, 24]. Furthermore, these behaviors [22] and influencing factors [14] differ between countries, and it is speculated that they may be influenced by genetic, environmental, cultural, and other factors. China is located in eastern Asia, and its genetics, geographical location, climate environment, and culture are very different from those in Europe. At present, several related studies exists in China, but they all focus on the relationship between single or several behaviors and obesity [25–28]. There are few studies from the perspective of energy balance. Only a few studies focus on primary school students in certain province or region [29–32], and national studies are few.

Elementary school students aged 10–12 years are in a critical growth and development period, and their behaviors are influenced by personal and family factors. To provide theoretical reference and practical guidelines for preventing and controlling overweight/obesity in school-aged children, this study compared the differences in EBRB of overweight/obese and normal weight primary school students to investigate key EBRB affecting their weight status, as well as major factors influencing their EBRB. The purpose is to provide a scientific basis for government functional departments to formulate national school sports health work strategies.

## Methods

### Participants

This is a cross-sectional study conducted in Northeast China, North China, Northwest China and Southwest China from March 2021 to July 2021. The sample size calculation was based on the formula “n = [Z^2^ α/2 p(1-p)] /δ^2^” used for cross-sectional studies. A previous study reported that the overweight/obesity rate of Chinese adolescents is 19% [6]; thus, δ is set as 0.19. Using α = 0.05, Zα/2 = 1.96, and δ = 0.03, the minimum sample size for this study was calculated to be 657. We stratified the study area according to geographical location and economic development; one province was selected from each of the Northeast, North China, Northwest, and Southwest regions. Eight administrative districts were selected from each province, and two primary schools were selected from each administrative district. Three classes were selected for grades 4–6 in each primary school (one class per grade). After excluding students with abnormal growth and development and major diseases, 4608 participants remained. Data from 4412 participants (2199 boys, 2213 girls) were included in the study after excluding participants with incomplete data and invalid questionnaires. This study was approved by the Ethics Committee of Shenyang Sport University (No. 202019) and was conducted according to the tenets of the Helsinki Declaration and its later amendments. All students participated voluntarily in this study, and we obtained written informed consent from their parents/guardians.

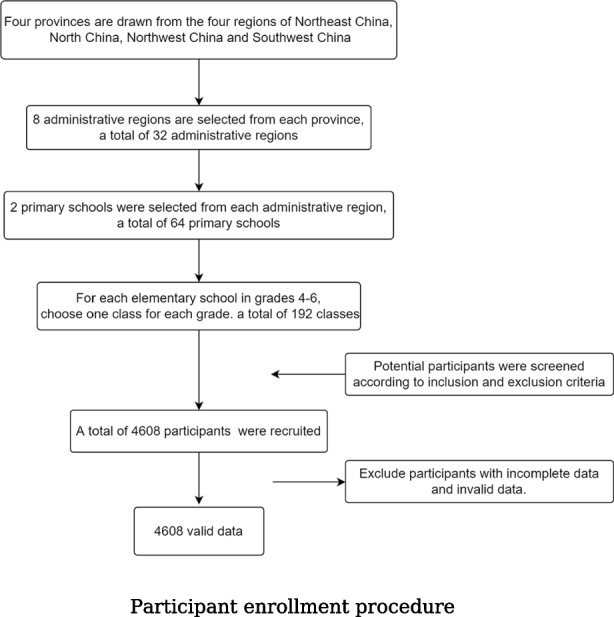



### Assessment

The study assessment comprised two parts, a body shape test and a questionnaire.

The children’s weight and height tests were conducted by dedicated researchers, and they are expressed as kilograms and centimeters, respectively, to one decimal point. The tests were carried out according to the measurement method in the “National Student Physical Health Standard (Revised in 2014)” (shown in Additional file 1: Appendix 1) promulgated by the Ministry of Education of the People’s Republic of China, and the student’s body shape is classified into obesity, overweight, normal weight, or underweight. Normal weight students were used as a reference group, while overweight and obese students were combined into the overweight/obese group. The number of underweight students was small, accounting for only 2.80% of the total sample; therefore, this group was not included in the present analysis.

A questionnaire was used to investigate the EBRBs of students and their influencing factors. The children’s EBRBs questionnaire was based on the European Energy balance Research to prevent excessive weight Gain among Youth (ENERGY) project: Design and methodology of the ENERGY cross-sectional survey “ [33], which has good reliability and validity [34]. The questionnaire was processed through localization, proofreading, and expert consultation and revised according to expert opinions and China’s actual national conditions. After modifying the questionnaire, the reliability and validity of the questionnaire were tested. The Cronbach α coefficient of the questionnaire was 0.734, and the KMO value was 0.910. Exploratory factor analysis could be carried out to extract two common factors, and the cumulative variance contribution rate was 56.148%, qualifying it for the investigation of EBRBs and influencing factors in Chinese school-aged children. The questionnaires were distributed on-site by professionally trained research team members, who instructed primary school students to complete them independently. The response rate of the questionnaires was 100%, and the effective rate was 95.75%.

The number of sugar-sweetened beverages per week reflects the sugary beverage behavior of elementary school students, while the number of weekly breakfasts obtained by adding the number of breakfasts on weekdays and weekends reflects the breakfast behavior of elementary school students. The weekly physical activity time obtained by adding up the time spent in sports reflects the physical activity of the primary school students, while the weekly screen time obtained by adding the screen-viewing time on weekdays and weekends reflects the screen viewing behavior of the primary school students.

Personal factors influencing EBRBs were set as attitudes, health beliefs, preferences, self-efficacy, and automaticity, while family factors included parental subjective norm, parent modeling, parental practices, home availability, and parental support. All variables were evaluated using the Likert 5-level scale. The questions, options, and scores for the influencing factors are shown in Additional file 2: Appendix 2.

### Data analysis

Data were tested for normality. Differences in EBRBs and influencing factors were compared between overweight/obese and normal weight primary school students using independent samples t-test. *P* values less than 0.05 were considered statistically significant. Taking the student’s weight class as the dependent variable (normal weight = “0”, overweight/obesity = “1”), number of sugary drinks per week, number of breakfasts per week, weekly physical activity time and weekly screen-viewing time as the independent variables, logistic regression analysis was carried out. Taking the number of breakfasts per week and the weekly screen-viewing time of overweight/obese students as dependent variables and their respective influencing factors as independent variables, linear regression analysis was carried out, respectively. All data were analyzed using SPSS 26.0 software.

## Results

A total of 4412 primary school students aged 10–12 years were investigated in this study. The number and proportion of each weight class are shown in Table 1. Among them, 1156 were overweight/obese primary school students and 3134 were normal weight primary school students. Referring to the “National Student Physical Health Standard (Revised in 2014),” the overweight/obesity rate was 26.2%. The average body mass index (BMI) of overweight/obese students were 23.42 ± 0.10 kg/m^2^, and the average BMI of normal weight students were 17.14 ± 0.03 kg/m^2^.

**Table 1:** Number and proportion of Chinese primary school students aged 10–12 in different weight grades (*n* = 4412)

Weight grade	Overall	Boys	Girls
obesity	525(11.9%)	297(13.5%)	228(10.3%)
overweight	631(14.3%)	335(15.2%)	296(13.4%)
normal weight	3134(71.0%)	1503(68.4%)	1631(73.7%)
under weight	122(2.8%)	64(2.9%)	58(2.6%)
overall	4412	2199	2213

### Differences in EBRBs between overweight/obese and normal weight students aged 10–12 years in China

Table 2 displays the results of the EBRB-related questionnaire and shows the differences in EBRBs between overweight/obese and normal weight students. No differences in beverage consumption and physical activity were observed between the two groups (*p* > 0.05); however, breakfast consumption was significantly lower in the overweight/obese group than in the normal weight group (*p* < 0.01). Further, the reported screen-viewing time was significantly higher in the overweight/obese group than in the normal weight group (*p* < 0.01). Differences in EBRBs according to sex were consistent with differences for each group at the overall level.

**Table 2:** Differences of EBRBs between overweight/obesity and normal weight Chinese primary school aged 10–12 years (*n* = 4290)

	Overall (*n* = 4290)			Boys (*n* = 2135)			Girls (*n* = 2155)		
	Normal weight	Overweight /Obesity	95%CI	Normal weight	Overweight /Obesity	95%CI	Normal weight	Overweight /Obesity	95%CI
	`X ± SE	`X ± SE		`X ± SE	`X ± SE		`X ± SE	`X ± SE	
Beverage behavior	1.17 ± 0.02	1.22 ± 0.04	(−0.046,0.132)	1.34 ± 0.04	1.30 ± 0.06	(− 0.170,0.094)	1.02 ± 0.03	1.11 ± 0.05	(−0.018,0.206)
Breakfast behavior	6.31 ± 0.02	6.09 ± 0.05^b^	(−0.325,-0.122)	6.26 ± 0.04	6.13 ± 0.06^a^	(−0.282,-0.003)	6.36 ± 0.03	6.06 ± 0.07^b^	(−0.456,-0.157)
Physical activity	4.43 ± 0.05	4.51 ± 0.08	(−0.107,0.278)	4.76 ± 0.08	4.67 ± 0.12	(−0.367,0.186)	4.12 ± 0.07	4.32 ± 0.12	(−0.065,0.467)
Video behavior	2.81 ± 0.05	3.26 ± 0.09^b^	(0.267,0.651)	3.18 ± 0.07	3.54 ± 0.12^a^	(0.082,0.639)	2.47 ± 0.06	2.93 ± 0.12^b^	(0.212,0.733)

^a^ means there is significant difference at 0.05 level, ^b^ means there is significant difference at 0.01 level

### Differences in EBRB influencing factors between overweight/obese and normal weight students aged 10–12 years in China

Differences in EBRB influencing factors between the overweight/obese and normal weight groups are shown in Table 3. The scores for beverage consumption influencing factors, “Health beliefs,” “Parental subjective norm,” “Parent modeling,” “Parental practices,” and “Home availability,” were significantly increased in the overweight/obese group compared to the normal weight group (*p* < 0.01 or *p* < 0.05). Overweight/obese boys’ scores for “Health beliefs,” “Self-efficacy,” “Parental subjective norm,” and “Parent modeling” were significantly increased compared to the normal weight boys’ scores (*p* < 0.01 or *p* < 0.05), while the overweight/obese girls’ scores for “Health beliefs,” “Parental subjective norm,” “Parent modeling,” and “Home availability” were significantly increased compared to the scores of normal weight girls (*p* < 0.01 or *p* < 0.05).

**Table 3:** Differences of EBRBS influencing factors between overweight/obesity and normal weight Chinese primary school aged 10–12 years in China (*n* = 4290)

			Overall (*n* = 4290)			Boys (*n* = 2135)			Girls (*n* = 2155)		
			Normal weight	Overweight /Obesity		Normal weight	Overweight /Obesity		Normal weight	Overweight /Obesity	
			`X ± SE	`X ± SE	95%CI	`X ± SE	`X ± SE	95%CI	`X ± SE	`X ± SE	95%CI
Beverage behavior	Personal variables	Health beliefs	3.85 ± 0.02	4.27 ± 0.03^b^	(0.353,0.490)	3.76 ± 0.03	4.22 ± 0.04^b^	(0.366,0.565)	3.93 ± 0.03	4.33 ± 0.04^b^	(0.300,0.490)
		Self-efficacy	4.11 ± 0.02	4.12 ± 0.03	(−0.055,0.081)	3.99 ± 0.03	4.12 ± 0.04^b^	(0.031,0.224)	4.21 ± 0.03	4.12 ± 0.04	(−0.189,0.007)
	Family environmental variables	Parental subjective norm	2.34 ± 0.02	2.48 ± 0.04^b^	(0.052,0.232)	2.35 ± 0.03	2.48 ± 0.06^b^	(0.023,0.270)	2.33 ± 0.03	2.48 ± 0.06^a^	(0.004,0.268)
		Parent modelling	2.47 ± 0.02	2.62 ± 0.04^b^	(0.069,0.223)	2.49 ± 0.03	2.60 ± 0.05^a^	(−0.009,0.208)	2.44 ± 0.03	2.65 ± 0.05^b^	(0.089,0.302)
		parental practices	2.50 ± 0.02	2.59 ± 0.04^a^	(0.011,0.166)	2.48 ± 0.03	2.56 ± 0.05	(−0.034,0.186)	2.51 ± 0.03	2.62 ± 0.05	(−0.004,0.219)
		Home availability	2.42 ± 0.02	2.56 ± 0.04^b^	(0.058,0.222)	2.45 ± 0.03	2.56 ± 0.05	(−0.011,0.218)	2.39 ± 0.03	2.57 ± 0.05^b^	(0.061,0.291)
Breakfast behavior	Personal variables	Attitude	4.67 ± 0.01	4.61 ± 0.02^a^	(−0.102,-0.010)	4.63 ± 0.02	4.59 ± 0.03	(−0.110,0.028)	4.70 ± 0.02	4.63 ± 0.03	(−0.125,-0.006)
		Health beliefs	3.37 ± 0.03	3.10 ± 0.04^b^	(−0.370,-0.182)	3.31 ± 0.04	3.06 ± 0.06^b^	(−0.376,-0.112)	3.43 ± 0.03	3.15 ± 0.06^b^	(−0.429,-0.162)
		Preference	4.65 ± 0.01	4.61 ± 0.02	(−0.089,0.012)	4.61 ± 0.02	4.62 ± 0.03	(−0.058,0.086)	4.68 ± 0.02	4.59 ± 0.03^a^	(−0.163,-0.019)
		Self-efficacy	4.63 ± 0.01	4.58 ± 0.02^a^	(−0.107,-0.006)	4.59 ± 0.02	4.60 ± 0.03	(−0.064,0.078)	4.67 ± 0.02	4.55 ± 0.03^b^	(−0.194,-0.049)
	Family environmental variables	Parental subjective norm	4.69 ± 0.01	4.60 ± 0.02^b^	(−0.135,-0.040)	4.64 ± 0.02	4.59 ± 0.03	(−0.108,0.029)	4.73 ± 0.02	4.60 ± 0.03^b^	(−0.201,-0.067)
		Parental support	4.64 ± 0.02	4.54 ± 0.03^b^	(−0.165,-0.044)	4.59 ± 0.02	4.53 ± 0.04	(−0.140,0.031)	4.69 ± 0.02	4.54 ± 0.04^b^	(−0.236,-0.064)
Physical activity	Personal variables	Health beliefs	3.88 ± 0.02	4.24 ± 0.04^b^	(0.294,0.456)	3.87 ± 0.04	4.26 ± 0.05^b^	(0.287,0.521)	3.88 ± 0.03	4.22 ± 0.05^b^	(0.230,0.455)
		Preference	4.62 ± 0.01	4.50 ± 0.02^b^	(−0.173,-0.068)	4.67 ± 0.02	4.54 ± 0.03^a^	(−0.198,-0.061)	4.56 ± 0.02	4.45 ± 0.04^b^	(−0.206,-0.045)
		Automaticity	4.48 ± 0.02	4.38 ± 0.03^b^	(−0.159,-0.038)	4.54 ± 0.02	4.41 ± 0.04^a^	(−0.203,-0.040)	4.42 ± 0.02	4.35 ± 0.04	(−0.177,0.001)
	Family environmental variables	Parental subjective norm	4.70 ± 0.01	4.73 ± 0.02	(−0.019,0.072)	4.67 ± 0.02	4.74 ± 0.03^a^	(0.004,0.132)	4.73 ± 0.02	4.72 ± 0.03	(−0.076,0.048)
Video behavior	Personal variables	Health beliefs	2.85 ± 0.03	3.06 ± 0.05^b^	(0.109,0.287)	2.78 ± 0.04	3.02 ± 0.07^b^	(0.108,0.364)	2.91 ± 0.04	3.11 ± 0.07^a^	(0.050,0.298)
		Self-efficacy	3.98 ± 0.02	4.01 ± 0.03	(−0.036,0.103)	3.90 ± 0.03	4.00 ± 0.04^a^	(0.003,0.201)	4.05 ± 0.03	4.02 ± 0.04	(−0.124,0.069)
	Family environmental variables	Parental subjective norm	2.24 ± 0.02	2.28 ± 0.03^a^	(0.017,0.170)	2.29 ± 0.03	2.32 ± 0.05^a^	(0.029,0.237)	2.19 ± 0.03	2.23 ± 0.05	(−0.065,0.160)
		parental practices	2.33 ± 0.02	2.46 ± 0.04^a^	(0.057,0.219)	2.40 ± 0.03	2.49 ± 0.05^a^	(0.028,0.253)	2.26 ± 0.03	2.44 ± 0.05^a^	(0.014,0.248)

^a^ means there is significant difference at 0.05 level, ^b^ means there is significant difference at 0.01 level

Factors influencing breakfast consumption, including scores for survey questions assessing “Attitude,” “Health beliefs,” “Self-efficacy,” “Parental subjective norm,” and “Parental support,” of overweight/obese students were significantly lower than those in the normal weight group (*p* < 0.01 or *p* < 0.05). Scores related to “Health beliefs” of obese/overweight boys were significantly lower than those of normal weight boys (*p* < 0.01). Obese/overweight girls’ scores related to “Health beliefs,” “Preferences,” “Self-efficacy,” “Parental subjective norm,” and “Parental support” were significantly lower than those of normal weight girls (*p* < 0.01 or *p* < 0.05).

Influencing factors of physical activity, including “Health beliefs” scores, were significantly higher in the overweight/obese group than in the normal weight group (*p* < 0.01), and the scores for items assessing “Preference” and “Autonomy” were significantly lower (*p* < 0.01). The scores of overweight/obese boys concerning “Health beliefs” and “Parental subjective norms” were significantly higher than those of normal weight boys (*p* < 0.01 or *p* < 0.05), while scores related to “Preference” and “Autonomy” were significantly lower (*p* < 0.05). The scores of overweight/obese girls related to “Health beliefs” were significantly higher than those of normal weight girls (*p* < 0.01), while the scores related to “Preference” were significantly lower (*p* < 0.01).

Influencing factors of screen-viewing behavior, including “Health beliefs,” “Parental subjective norms,” and “Parental practices,” were significantly higher in overweight/obese students than in the normal weight group (*p* < 0.01 or *p* < 0.05). Overweight/obese boys’ scores related to “Health beliefs,” “Self-efficacy,” “Parental subjective norms,” and “Parental practices” were significantly higher than those of normal weight boys (*p* < 0.01 or *p* < 0.05). Overweight/obese girls’ scores related to “Health beliefs” and “Parental practices” were significantly higher than those of normal weight girls (*p* < 0.05).

### Relationship between overweight/obesity and EBRB in Chinese primary school students aged 10–12 years

Logistic regression analysis was used to analyze the relationship between overweight/obesity and EBRBs (beverage consumption behavior, breakfast consumption behavior, physical activity, and screen-viewing behavior). Results are shown below in Table 4. There is no multicollinearity among the independent variables. Breakfast consumption behavior (odds ratio OR: 0.911, confidence interval CI: 0.870–0.954) and screen-viewing behavior (OR: 1.055, CI: 1.030–1.080) were independent factors affecting overweight/obesity. Regular breakfast consumption behavior was associated with a reduced risk of overweight/obesity, and increasing the number of breakfasts consumed by one additional breakfast per week reduced the risk of overweight/obesity by 9%; in girls, the risk was reduced by 13.3%. Increased screen-viewing time was associated with an increased risk of overweight/obesity; for every additional hour of screen time per week, the risk of overweight/obesity increased by 5.5%,4.2% for boys, 6.9% for girls.

**Table 4:** Results of EBRBs Logistic regression analysis between overweight/obesity and primary school students aged 10–12 years in China (*n* = 4290)

	Overall (*n* = 4290)				Boys (*n* = 2135)				Girls (*n* = 2155)			
	B	*p*	OR	95%CI	B	*p*	OR	95%CI	B	*p*	OR	95%CI
Breakfast behavior	−0.094	0.000	0.911	0.870–0.954					−0.143	0.000	0.867	0.810–0.928
Video behavior	0.053	0.000	1.055	1.030–1.080	0.041	0.000	1.042	1.010–1.074	0.066	0.001	1.069	1.028–1.111
constant	−0.578	0.000	0.561		−1.003	0.000	0.367		−0.428	0.063	0.652	

B represents the non-standardized coefficient, P represents the significance, OR represents the odds ratio and 95%CI represents the 95% confidence interval

### Relationship between breakfast consumption behavior and influencing factors among overweight/obese primary school students aged 10–12 years in China

With the number of breakfasts per week for overweight/obese primary school students as the dependent variable and personal factors (attitude, health beliefs, preference, self-efficacy, and automaticity) and family factors (parental subjective norm, parent modeling, parental practices, home availability, and parental support) as the independent variables, multiple linear regression analysis was performed. The results showed in Table 5 that the regression model was significant (*F* = 45.775, *p* < 0.01), and there was no multicollinearity among the respective variables. Home availability, self-efficacy, preference, parent modeling, and attitude, were all positively correlated with the weekly number of breakfasts.

**Table 5:** The results of linear regression analysis between breakfast consumption and influencing factors of overweight/obese primary school students aged 10–12 years in China (*n* = 1156)

	Overall (*n* = 1156)					Boys (*n* = 632)					Girls (*n* = 524)				
		B(SE)	β	*p*	VIF		B(SE)	β	p	VIF		B(SE)	β	p	VIF
	Constant	1.09(0.35)		0.002		Constant	0.76(0.41)		0.065		Constant	1.55(0.52)		0.003	
Personal variables	Self-efficacy	0.28(0.07)	0.14	0.000	1.743	Self-efficacy	0.50(0.09)	0.25	0.000	1.647	Preference	0.21(0.09)	0.10	0.024	1.136
	Preference	0.23(0.08)	0.11	0.002	1.822	Preference	0.28(0.01)	0.14	0.004	1.867	Health beliefs	0.11(0.05)	0.09	0.034	1.011
	Attitude	0.15(0.08)	0.07	0.044	1.505	Attitude	0.19(0.09)	0.10	0.028	1.495					
Family environmental variables	Home availability	0.28(0.05)	0.18	0.000	1.184	Home availability	0.20(0.06)	0.13	0.001	1.149	Home availability	0.41(0.07)	0.25	0.000	1.131
	Parent modelling	0.17(0.05)	0.09	0.002	1.177						Parent modelling	0.33(0.08)	0.17	0.000	1.104

B represents the unstandardized coefficient, SE represents the standard error, P represents the significance, VIF represents the collinearity

The regression model of boys’ breakfast behavior was significant (*F* = 44.881, *p* < 0.01), and there was no multicollinearity among the independent variables. Self-efficacy, preference, home availability, and attitude were all positively correlated with the weekly number of breakfasts.

In addition, the regression model of girls’ breakfast behavior was significant (*F* = 22.658, *p* < 0.01), and there was no multicollinearity among the independent variables. Home availability, parent modeling, preference, and health beliefs were all positively correlated with the weekly number of breakfasts.

### Relationship between screen viewing behavior and influencing factors among overweight/obese primary school students aged 10–12 years in China

Multiple linear regression was performed with the weekly screen viewing time of overweight/obese primary school students as dependent variables, and personal factors (attitude, health beliefs, preference, self-efficacy, and automaticity) and family factors (parental subjective norm, parent modeling, and parental practices) as independent variables. The results showed in Table 6, the regression model was significant (*F* = 31.874, *p* < 0.01), and there was no multicollinearity among the respective variables. Preference, parental practices, and parental subjective norm were all positively correlated with weekly screen viewing time, while self-efficacy was negatively correlated with weekly screen viewing time.

**Table 6:** Results of linear regression analysis on the screen viewing activities and influencing factors of overweight/obese primary school students aged 10–12 years in China (*n* = 1156)

	Overall					Boys					Girls				
		B(SE)	β	p	VIF		B(SE)	β	p	VIF		B(SE)	β	p	VIF
	Constant	2.06(0.59)		0.000		Constant	2.65(0.75)		0.000		Constant	1.52(0.87)		0.082	
Personal variables	Preference	0.50(0.09)	0.19	0.000	1.15	Preference	0.63(0.13)	0.237	0.000	1.156	Attitude	0.46(0.18)	0.143	0.010	1.319
	Self-efficacy	−0.44(0.10)	−0.15	0.000	1.07	Self-efficacy	−0.50(0.14)	−0.164	0.000	1.041	Self-efficacy	−0.39(0.14)	−0.141	0.006	1.122
Family environmental variables	Parental practices	0.34(0.10)	0.13	0.000	1.261	Parental practices	0.36(0.13)	0.133	0.005	1.113	Parental practices	0.38(0.13)	0.148	0.005	1.192
	Parental subjective norm	0.26(0.12)	0.08	0.025	1.263						Parent modelling	0.36(0.15)	0.121	0.021	1.185

B represents the unstandardized coefficient, SE represents the standard error, P represents the significance, VIF represents the collinearity

The results of multiple linear regression analysis of boys’ screen viewing behavior showed that the regression model was significant (*F* = 23.214, *p* < 0.01), and there was no multicollinearity among the independent variables. Self-efficacy was negatively correlated with weekly screen viewing time, while preference and parental practices were positively correlated with weekly screen viewing time.

The results of multiple linear regression analysis of girls’ screen viewing behavior showed that the regression model was significant (*F* = 15.300, *p* < 0.01), and there was no multicollinearity among the independent variables. Attitude, parental practices, and parent modeling were positively correlated with weekly screen viewing time, while self-efficacy was negatively correlated with weekly screen viewing time.

## Discussion

This study pooled 4412 school-aged children aged 10–12 years old from four regions: Northeast China, North China, Northwest China, and Southwest China. The overweight/obesity rate of primary school students aged 10–12 years in the present study was 26.2%: boys, 28.7% and girls, 23.7%. Both boys and girls had lower rates of overweight/obesity than European children of the same age [22]; but higher than the overall rate of Chinese teenagers [6]. Compared with normal weight primary school students, the overweight/obese students had significantly reduced frequency of breakfast and significantly increased screen viewing time. The overweight/obese students and normal weight students showed differences in individual and family factors of beverage consumption behavior, breakfast consumption behavior, and screen viewing behavior, as well as individual factors of physical activity. The unhealthy breakfast and screen viewing behavior of overweight and obese primary school students are a consequence of the combined effect of their personal and family factors.

Regular consumption of high-quality breakfast has positive effects on child growth and development [35] and cognition and learning [36], but school-aged children often skip breakfast [37]. In this study, 71.8% of normal weight students reported that they consumed breakfast every day, while only 66.6% of overweight/obese students reported daily breakfast consumption. Further, the breakfast consumption frequency of normal weight students was significantly higher than that of overweight/obese students. Regular breakfast consumption behavior is related to reduced overweight/obesity risk, whereas skipping breakfast is associated with an increased risk. This result is counterintuitive; from an energy balance perspective, it seems that skipping breakfast would result in decreased energy intake, reducing the risk of overweight/obesity. However, skipping breakfast is associated with an increased risk of overweight/obesity [38–40]. Indeed, studies have shown that while skipping breakfast reduces total energy intake [41], it is also correlated with reduced physical activity [42] and total energy expenditure [43]; therefore, the reduction in energy expenditure caused by skipping breakfast may be the reason for overweight/obesity. Another study showed that skipping breakfast caused a compensatory increase in energy intake at lunch and dinner, which led to the total daily energy intake not only not decreasing, but even increasing [44]. Therefore, skipping breakfast increases the risk of overweight/obesity. Our findings agree with previous results that indicate an association between skipping breakfast and an increased risk of overweight/obesity.

Attitude, preference, and self-efficacy are important personal factors that influence breakfast consumption by overweight/obese primary school students. Compared with normal weight students, overweight/obese students have lower scores in attitude and self-efficacy, which implies that their intention and perceived control of breakfast behaviors are lower, leading to reduced frequency of breakfast consumption. Studies have shown that parents can help prevent unhealthy eating behaviors in their children [45], and that if parents regularly consume breakfast and provide breakfast to their children, they can effectively prevent their children from skipping breakfast [46]. The present study demonstrates that parental modeling and sufficient availability of breakfast at home are important family factors that promote regular breakfast consumption in school-aged children. However, we did not find any differences in parental modeling and home availability between overweight/obese and normal weight students. Thus, it can be concluded that the irregular breakfast consumption behaviors of overweight/obese students are mainly related to individual factors, while the influence of family factors is relatively weak. However, the influence of family factors may not be limited to direct effects. Therefore, this study conducted a correlation analysis between family and individual factors. The results revealed a certain correlation between family availability and parent modeling and self-efficacy, preference, attitude, and health beliefs (shown in Additional file 3: Appendix 3). Family factors may also indirectly affect students’ breakfast consumption behaviors by influencing individual factors. Together, these results suggest that irregular breakfast consumption behavior is the key EBRB that influences the risk of overweight/obesity in primary school students, and although unhealthy breakfast consumption behavior is the combined effect of individual and family factors, individual factors have a greater influence on this risk.

Sedentary behavior refers to any conscious behavior with an energy consumption level ≤ 1.5 METs, when carried out in a sitting or leaning posture [47]. Screen-viewing activities (e.g., watching television, playing videogames, and browsing the internet) are a common form of sedentary behavior among primary school students. In this study, the weekly screen-viewing time of overweight/obese students was significantly higher compared to that of normal weight students. Furthermore, the weekly screen-viewing time was positively correlated with overweight/obesity. This is similar to the findings of Li et al. [48]; in their study among overweight and obese children aged 8–14 years who showed a positive correlation between BMI, fat percentage, and leisure screen time. Furthermore, numerous studies have also shown ​that long-term weekly screen-viewing time behavior is associated with an increased risk of overweight/obesity in school-aged children [12, 49–51].

Preference and self-efficacy are important personal factors influencing screen-viewing behavior. However, no difference in personal factors was observed between overweight/obese and normal weight primary school students. Parental practices and parental subjective norms are important family factors that influence screen-viewing behavior of overweight/obese primary school students. Studies have shown that strict family rules [52] and good parental role modeling [53] can effectively control children’s screen-viewing behavior. In this study, overweight/obese primary school students receive more attention and more lax control from their parents, which leads to their longer screen viewing behavior than normal-weight primary school students. In addition, family factors, such as parental subordinate norms, parent modeling, and parental practices are related to individual factors, such as attitude, preferences, and autonomy (shown in Additional file 4: Appendix 4). This indicates that family factors not only directly affect screen-viewing activities in children but also indirectly affect screen-viewing activities by influencing children’s individual factors. Together, these results suggest that long-term screen-viewing behavior is a key EBRB that influences the risk of overweight/obesity in primary school students, although this adverse EBRB is also a result of the combined effect of individual and family factors, the role of family factors is more important.

Drinking sugar-sweetened beverages is reported to be strongly associated with overweight/obesity [54]. However, in this study, no difference in the beverage consumption behavior of overweight/obese primary school students and normal weight students was observed. The subjects in this study were young and had poor understanding of the measurement units. This study used a relatively vague frequency to describe the number of sugar-sweetened beverages per week and failed to adopt precise measurement units. This may have caused that there was no apparent difference in beverage consumption behavior between the two groups of students.

Although study have also shown that low levels of physical activity [11] are closely related to overweight/obesity, no difference in physical activity between the overweight/obese primary school students and the normal weight students was observed. Regarding the influencing factors, health beliefs, preference, and automaticity were found to have their own advantages and disadvantages, but family factors were of great significance to physical activity [55, 56]. It is possible that the combined effect of these factors on the physical activity levels of the two groups had no substantial difference.

### Suggestion

Children aged 10–12 years are in a critical period of growth and behavior establishment. It is suggested that children’s affection for breakfast should be improved through dietary education, and parents should set a good breakfast example, provide necessary breakfast guarantee, and gradually establish children’s healthy breakfast behavior. At the same time, parents are advised to restrict children’s long-term screen-viewing behavior through strict control and clear family rules. Finally, establishing good behaviors related to energy balance can help control the overweight and obesity of school-aged children. In addition, the influence of school factors on school-aged children’s EBRB cannot be ignored, it is suggested that follow-up research should focus on the influence of school factors on the children’s EBRB.

### Limitations

This study has certain limitations. As a cross-sectional and retrospective study, there is an unavoidable retrospective bias. The survey was conducted using questionnaires, and the results were subject to a certain degree of subjective bias. Although it was confirmed that personal factors, family factors, and EBRB were correlated, a mediation effect test was not conducted. For the external factors affecting EBRB in overweight/obese primary school students, this study involved only families and did not consider the influence of school. In addition, because the exogenous variables of pupils and parents were not included in this study, the results of this study have some limitations.

## Conclusions

Irregular breakfast consumption and excessive screen viewing time engagement are key EBRB associated with overweight/obesity among Chinese primary school students aged 10–12 years. The unhealthy breakfast consumption and screen viewing activities of overweight/obese primary school students are the result of a combination of personal and family factors.

## Supplementary Information

**Additional file 1.**

**Additional file 2.**

**Additional file 3.**

**Additional file 4.**

## Data Availability

The datasets used and analyzed during the current study cannot be shared at this time as the data also forms part of an ongoing study，If there is any need, please contact the author (Professor Bo CHANG - email address: changbo8387@163.com).

## Abbreviation

EBRB: energy balance-related behavior
