# Supplementary material for: Relationship between energy balance-related behaviors and personal and family factors in overweight/obese primary school students aged 10–12 years in China: a cross-sectional study
Source: BMC Public Health. 2022 Oct 27;22:1968. doi: 10.1186/s12889-022-14238-x (PMC9608935; doi:10.1186/s12889-022-14238-x)
Supplement: Supplementary file 1 — Additional file 1. [file 12889_2022_14238_MOESM1_ESM.pdf]

Chinese 10-12-year-old primary school students BMI grade evaluation standard (kg/m^2)

	boys			girls		
	Grade four	Grade five	Grade six	Grade four	Grade five	Grade six
normal weight	14.2~20.1	14.4~21.4	14.7~21.8	13.7~19.4	13.8~20.5	14.2~20.8
underweight	≤14.1	≤14.3	≤14.6	≤13.6	≤13.7	≤14.1
overweight	20.2~22.6	21.5~24.1	21.9~24.5	19.5~22.0	20.6~22.9	20.9~23.6
obesity	≥22.7	≥24.2	≥24.6	≥22.1	≥23.0	≥23.7

Source: National Student Physical Health Standard (revised in 2014) issued by the Ministry of Education of the People's Republic of China
