# Supplementary material for: Relationship between energy balance-related behaviors and personal and family factors in overweight/obese primary school students aged 10–12 years in China: a cross-sectional study
Source: BMC Public Health. 2022 Oct 27;22:1968. doi: 10.1186/s12889-022-14238-x (PMC9608935; doi:10.1186/s12889-022-14238-x)
Supplement: Supplementary file 2 — Additional file 2. [file 12889_2022_14238_MOESM2_ESM.pdf]

The influencing factors topics, options and scores of energy balance-related behaviors

		Subject	Option
Personal variables	Attitude	I think that drinking fizzy drinks is/ eating breakfast is/ physical activity is/ watching TV is···	very good (5), very bad (1)
	Health beliefs	I think that drinking fizzy drinks / NOT eating breakfast / NOT doing physical activities/ watching too much television will make me fat.	very agree (5), very disagree (1)
	Preference	I like the taste of fizzy drinks / eating breakfast /doing physical activity/ watching television.	very agree (5), very disagree (1)
	Self-efficacy	I find not drinking fizzy drinks / eating breakfast every day/ doing physical activity everyday/ NOT watching television...	very simple (5), very difficult (1)
	Automaticity	Drinking fizzy drinks/ Eating breakfast / Doing physical activity/ Watching television is something I do without even really thinking about it	very agree (5), very disagree (1)
Family environmental variables	Parental subjective norm	If you drink fizzy drinks/eat breakfast/do physical activity/watch television ,your parents think this is···	very good (5), very bad (1)
	Parent modelling	How often do your parents drink fizzy drinks/eat breakfast/do physical activity/watch television?	always (5), never (1)
	Parental practices	If you ask your parents for a fizzy drink , you get one. /If you ask your parents to buy a certain band of food or drink for breakfast, will he(she) do it? /If you indicate that you like a certain physical activity， will your parents allow you to do it?/If you ask your parents to watch television, you can do so.	always (5), never (1)
	Home availability	Are there usually fizzy drinks/breakfast products at your home?	always (5), never (1)
	Active encouragement	Your parents encourage you to have breakfast/ do physical activity.	Very agree (5), very disagree (1)
