# Supplementary material for: Relationship between energy balance-related behaviors and personal and family factors in overweight/obese primary school students aged 10–12 years in China: a cross-sectional study
Source: BMC Public Health. 2022 Oct 27;22:1968. doi: 10.1186/s12889-022-14238-x (PMC9608935; doi:10.1186/s12889-022-14238-x)
Supplement: Supplementary file 3 — Additional file 3. [file 12889_2022_14238_MOESM3_ESM.pdf]

Correlation between personal and family factors in breakfast behavior of overweight/obese primary school students aged 10-12 in China

	overall（n=1156）			boys（n=632）			girls（n=524）	
	Self-efficacy	Preference	Attitude	Self-efficacy	Preference	Attitude	Preference	Health beliefs
Home availability	0.314**	0.298**	0.247**	0.315**	0.317**	0.24**	0.272**	0.025
Parent modelling	0.323**	0.278**	0.204**				0.231**	-0.013

Note: * means significant correlation at 0.05 level ,** means significant correlation at 0.01 level.
