# Supplementary material for: Relationship between energy balance-related behaviors and personal and family factors in overweight/obese primary school students aged 10–12 years in China: a cross-sectional study
Source: BMC Public Health. 2022 Oct 27;22:1968. doi: 10.1186/s12889-022-14238-x (PMC9608935; doi:10.1186/s12889-022-14238-x)
Supplement: Supplementary file 4 — Additional file 4. [file 12889_2022_14238_MOESM4_ESM.pdf]

Correlation between personal factors and family factors in Chinese 10-12-year-old overweight/obese primary school students' video-screening behavior

	overall（n=1156）		boys（n=632）		girls（n=524）	
	Preference	Self-efficacy	Preference	Self-efficacy	Attitude	Self-efficacy
Parental practices	0.238**	0.016	0.213**	0.093*	0.359**	-0.07
Parental subjective norm	0.184**	0.009				
Parent modelling					0.347**	-0.055

Note: * means significant correlation at 0.05 level ,* * means significant correlation at 0.01 level.
